# Understanding communication networks in the emergency department

**DOI:** 10.1186/1472-6963-9-247

**Published:** 2009-12-31

**Authors:** Nerida Creswick, Johanna I Westbrook, Jeffrey Braithwaite

**Affiliations:** 1 Health Informatics Research and Evaluation Unit, Faculty of Health Sciences, The University of Sydney, Australia; 2 Centre for Clinical Governance Research, Australian Institute of Health Innovation, Faculty of Medicine, University of New South Wales, Australia

## Abstract

**Background:**

Emergency departments (EDs) are high pressure health care settings involving complex interactions between staff members in providing and organising patient care. Without good communication and cooperation amongst members of the ED team, quality of care is at risk. This study examined the problem-solving, medication advice-seeking and socialising networks of staff working in an Australian hospital ED.

**Methods:**

A social network survey (Response Rate = 94%) was administered to all ED staff (n = 109) including doctors, nurses, allied health professionals, administrative staff and ward assistants. Analysis of the network characteristics was carried out by applying measures of density (the extent participants are concentrated), connectedness (how related they are), isolates (how segregated), degree centrality (who has most connections measured in two ways, in-degree, the number of ties directed to an individual and out-degree, the number of ties directed from an individual), betweenness centrality (who is important or powerful), degree of separation (how many ties lie between people) and reciprocity (how bi-directional are interactions).

**Results:**

In all three networks, individuals were more closely connected to colleagues from within their respective professional groups. The problem-solving network was the most densely connected network, followed by the medication advice network, and the loosely connected socialising network. ED staff relied on each other for help to solve work-related problems, but some senior doctors, some junior doctors and a senior nurse were important sources of medication advice for their ED colleagues.

**Conclusions:**

Network analyses provide useful ways to assess social structures in clinical settings by allowing us to understand how ED staff relate within their social and professional structures. This can provide insights of potential benefit to ED staff, their leaders, policymakers and researchers.

## Background

Emergency departments (EDs) are complex health care settings, characterised as high pressure, high intensity environments in which it can be stressful to work[1,2]. Work in EDs requires collaboration among health care workers from different professions in delivery of care to patients, with frequent interaction among staff to communicate patient and associated information[3].

Communication in EDs is complex and mainly face-to-face[4], and disjointed[5]. As in other health care settings, work patterns are often professionalised and tribal[6,7]. Staff carry out organisational work and clinical work simultaneously in order to manage and deliver patient care[8] and they relate to most other departments within the hospital to accomplish treatment and placement of patients if they are to be admitted or returned to the community[9-11].

Efficient communication and effective interpersonal interactions are vital in most organisational endeavours, but particularly in demanding environments. Without good communication and cooperation amongst members of an ED team, people will lack vital information. Work organisation will suffer, and this will likely lead to poor quality of care and the propensity for greater errors[12], with the potential to affect not only the ED but the rest of the hospital. Poor communication and coordination have been identified as research priorities for improving patient safety in developed countries[13].

The aim of this study was to use social network analysis to measure communication patterns and staff interactions within an ED. In order to accomplish this, three aspects of communication and interaction amongst ED staff were examined: problem-solving, medication advice-seeking and socialising. The problem-solving network is a general network which encompasses clinical and organisational interactions; the medication-advice seeking network involves key interactions of a clinical nature; and the socialising network describes informal interpersonal relationships which in turn influence work-related interactions.

## Methods

A social network survey was administered to all 109 ED staff working in an Australian metropolitan teaching hospital. In order to identify the interactions between staff, a comprehensive network approach was taken. We acquired a list of names of all staff working in the ED at the time of the survey. Using this list, survey respondents (n = 103, Response Rate = 94%) answered questions regarding: from whom they sought help to solve work-related problems, from whom they sought advice for medication decisions and tasks, and with whom they socialised at work. Problem solving, advice seeking, and social relationships are typical relationships measured in network studies of organisations[14]. The social network questionnaire was designed with reference to standard social network questions used in other studies[15-22]. Specific questions regarding medication advice-seeking interactions have not been previously considered, and these interactions were selected as objects of study because of the planned introduction to the hospital of an electronic medication management system. Complementing the examination of the networks, doctors, nurses and allied health professionals (n = 95) were asked to respond to two additional survey items about medication-related communication using a 5-point Likert scale. This was designed to allow us to compare the network results with information about health professionals' perceptions of communication around medication. Administrative staff were not given these two questions to complete. Demographic data were collected, including participants' profession and length of experience in profession.

In social network analyses, social structure is described in terms of nodes (individual clinicians and support staff in this study) and ties (the relationships between them). Description of the network data involved visual analysis of network diagrams produced using NetDraw[23]. This software converts matrices of network data into diagrams with nodes (or individuals) positioned optimally using complex algorithms. Analysis of the network characteristics was carried out by applying measures of connectedness (how densely participants are linked), isolates (how segregated), degree centrality (who has most connections measured in two ways, in-degree, the number of ties directed to an individual and out-degree, the number of ties directed from an individual), betweenness centrality (who is important or powerful), degree of separation (how many ties lie between people, or, the number of ties on the shortest route connecting two nominated individuals, calculated in two ways, as an average and as a maximum) and reciprocity (how bi-directional are interactions) for the three networks. These measures were calculated using the most widely used network analysis software, UCINET v6[24,25].

## Results

### Problem-solving network

**Figure 1:**
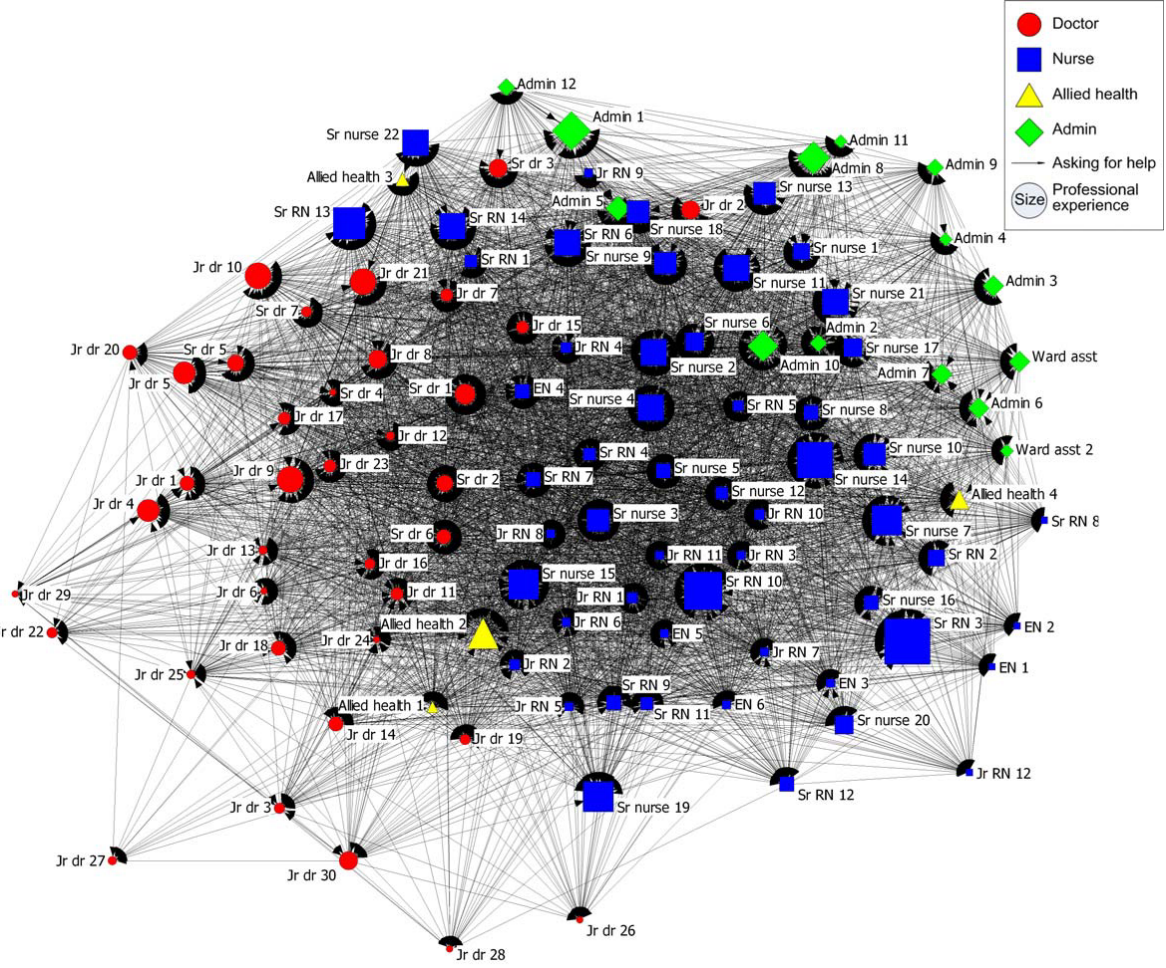
**Work-related problem solving network**.

When ED staff require help to solve work-related problems they ask colleagues from within their own profession, as indicated by the colour and shape of nodes in Figure 1. Most participants are positioned adjacent to those from their own profession. Most nurses are located in the centre and right-hand side of the network, most doctors in the left side of the network, the allied health professionals are located throughout the network and most administrative staff are positioned in the top right quarter of the network. Those in the centre of the network ask more people or are asked by more people for help to solve a work-related problem. They are more active in working with others to find solutions than those on the periphery.

Staff with a range of experiences are located throughout most of the problem-solving network as indicated by the range of sizes of the nodes throughout the network in Figure 1. However, many of the junior doctors are located in close proximity to each other, peripherally, on the left of the network. Less experienced medical staff seem thus to rely on each other rather than ask more senior staff for help to solve work-related problems.

This network is relatively highly connected with 53% of all possible ties represented (Connectedness = 53%, Table 1), namely given all the possible ties between individuals, 53% of these are reported for the work-related problem solving network. There are more ties within some of the professional groups than overall, with 69% of ties present between nurses. Only 45% of ties are represented between doctors. There are only 17% of ties present between allied health professionals, who instead tend to seek help from nurses and doctors.

**Table 1:** Summary of problem-solving, medication advice and socialising network characteristics

	Work-related problem solving network	Medication advice-seeking network	Socialising network
**Connectedness (Density of whole network)**	53%	37%	18%

**Profession with high density within professional group**	Admin (74%)	Nurses (58%)	Admin (49%)

**High densities of interaction between professional groups**	Doctors to Allied health (46%)	Nurses to Doctors (55%)	Admin to Nurses (21%)

**Degree of separation (Average for the network)**	1 to 2 degrees of separation	1 to 2 degrees of separation	1 to 2 degrees of separation

**Largest degree of separation**	3	4	7

**Reciprocity**	43%	26%	24%

**High in-degree**	Sr drs & Sr nurses	Sr drs, Jr drs & Sr nurse	Sr nurses, Sr RNs, Jr RNs, EN

**High out-degree**	Sr dr, Sr nurses, Sr RN, Jr RNs & EN	Sr nurses, Sr RNs, Jr RNs & Admin	Jr drs, Sr nurses, Sr RNs, Jr RNs, EN & Admin

**High betweenness**	Sr drs & Sr nurses	Sr drs, Jr dr, Sr nurses & Sr RNs	Jr drs, Sr nurses, Sr RNs, EN & Admin

With 43% of ties reciprocated (Reciprocity = 43%, Table 1), ie both parties agreed that they sought help from each other, there are horizontal as well as hierarchical aspects to the structure of the network. Although there are particular individuals being asked for help by other members in the unit, assistance is also given and received amongst many of the staff. The senior doctors and senior nurses provide help to a large number of the ED staff. Senior doctor 4 has the greatest number of staff members asking him or her for help to solve work-related problems, with 92 people reporting Senior doctor 4 as a source of help (in-degree = 92). Three other senior doctors and seven senior nurses also have a large number of other people asking them for advice, as indicated by their high in-degree centralities (Table 1). Senior doctor 6 is the most powerful actor, with the highest betweenness centrality value (Betweenness centrality = 280.9), indicating that they are sitting on many of the shortest routes between other members of the unit. In essence this demonstrates that much communication passes through them and that they communicate with many people. Two other senior doctors and seven senior nurses are also dominant in the network. They may be controlling information related to solving work-related problems or have the potential to do so, and have capacity to help others in solving work-related problems.

Help is readily available in this unit, shown by the high level of connectedness with an average of only one to two degrees of separation between any pair of nodes in the network, indicating that on average ED staff are either directly connected to each other or only one other person is between each pair of ED staff members (degree of separation = 1 to 2, Table 1), with no individual more than three degrees of separation from any other (maximum degree of separation = 3, Table 1). There are no isolates in this network, indicating that all ED staff members are involved in solving problems.

### Medication advice-seeking network

**Figure 2:**
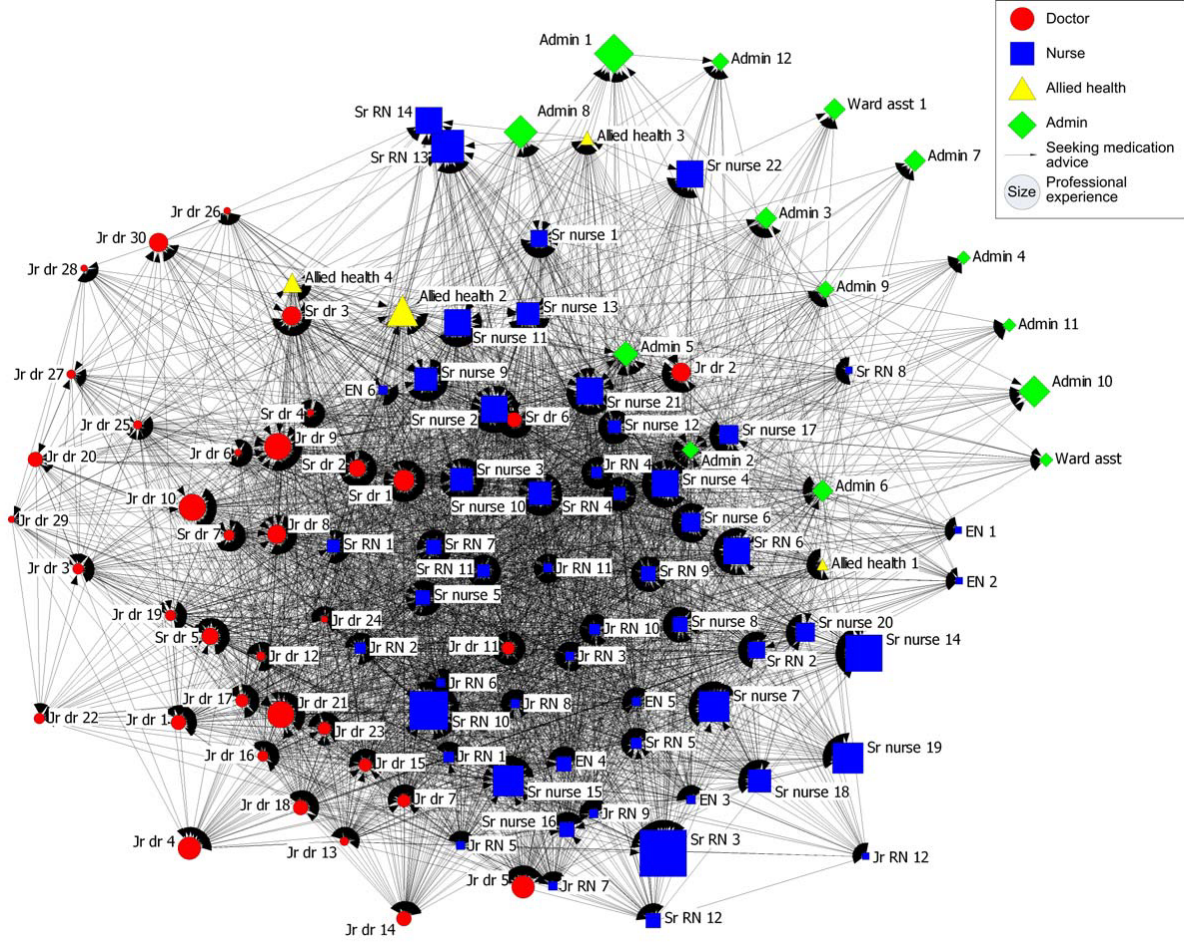
**Medication advice-seeking network**.

As in the problem-solving network, some of the senior doctors play a central role in the medication advice-seeking network. This is shown in Figure 2, where Senior doctors 1, 2 and 6, and Junior doctor 11 and Junior doctor 24 are more centrally located than their other medical colleagues, and in Table 1, where Senior doctors and Junior doctors have high in-degrees.

The medication advice-seeking network is moderately connected with 37% of all possible ED staff connections present (Density of whole network = 37%, Table 1). Sources of medication advice are readily available with each individual no more than four degrees of separation from any other (Maximum degree of separation = 4, Table 1), and they average only one to two degrees of separation from each other (Degree of separation = 1 to 2, Table 1). The ties between the nurses are the most dense (58%, Table 1). Ties between doctors are also quite dense (37%). The ties between allied health professionals are less dense (25%), as are those between administrative staff (26%). There were no isolates in this network.

Having only 26% of medication advice-seeking ties reciprocated (Reciprocity = 26%, Table 1) indicates the importance of particular individuals in the network with high in-degree centralities such as Senior doctor 1 (in-degree = 77), Senior doctor 2 (in-degree = 80), Senior doctor 5 (in-degree = 65), Senior doctor 6 (in-degree = 76) and Senior doctor 7 (in-degree = 66), Junior doctor 6 (in-degree = 63), Junior doctors 8 and 9 (in-degree = 64) and Senior nurse 2 (in-degree = 68). They are people on whom many other ED staff depend to provide advice regarding medication decisions and tasks. They seek or are sought by more individuals for medication advice, as well as by more nurses than their other medical colleagues. Junior doctor 2 is located at some distance from other doctors, indicating that he or she asks or is asked for help by more administrative staff and nurses than the other doctors.

### Attitudes to medication advice seeking and communication between doctors and nurses

More staff agreed (42.1%) than disagreed (34.8%) with the statement that doctors often seek advice from nurses about prescribing decisions (Table 2). Many of those who disagreed were doctors located peripherally from the centre of the medication advice network and separated from nurses. Many of these staff were junior doctors located moderately peripherally in the network, indicating that they do not interact with a lot of nurses. Most staff (67.9%) agreed that if doctors and nurses talked more frequently, there would be fewer medication errors, and only four staff disagreed (Table 2). No staff members strongly disagreed that if doctors and nurses talked more frequently, there would be fewer medication errors.

**Table 2:** Perceptions of ED clinical staff (n = 95) regarding communication between doctors and nurses

	Strongly disagree N (%)	Disagree N (%)	Un-certain N (%)	Agree N (%)	Strongly agree N (%)	No response N (%)
**Doctors often seek advice from nurses about prescribing decisions**	7 (7.4)	26 (27.4)	15 (15.8)	34 (35.8)	6 (6.3)	7 (7.4)

**If doctors and nurses talked more frequently, there would be fewer medication errors**	0 (0.0)	4 (4.2)	10 (10.5)	42 (44.2)	32 (33.7)	7 (7.4)

### Socialising network

**Figure 3:**
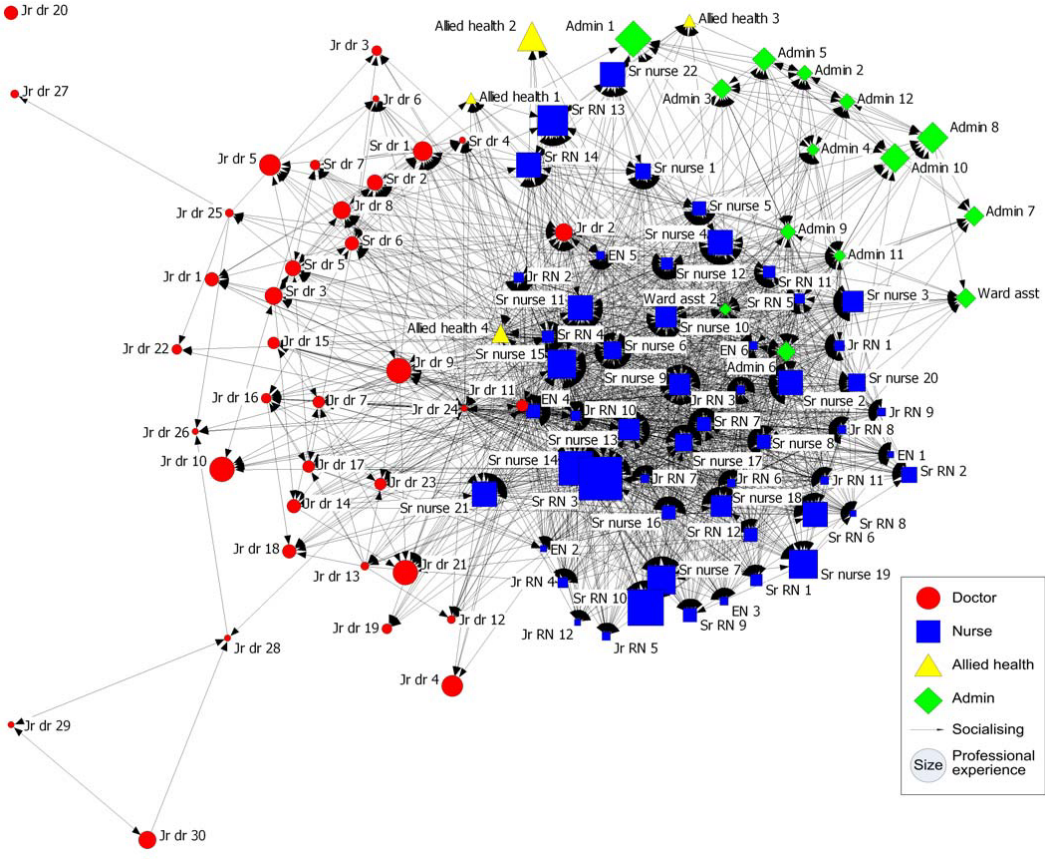
**Socialising network**.

ED staff largely socialise tribally, ie with colleagues from within their own profession (Figure 3). However, Junior doctors 2, 11 and 24 are located more centrally than other doctors, and Allied health professional 4 is located more centrally and apart from other allied health professionals indicating that these individuals also socialise with those from different professions.

Individuals with a variety of experience are located throughout the socialising network in the ED. This network is sparsely connected, with only 18% of all possible ties between people present (Connectedness = 18%, Table 1). This is the lowest density of the three networks examined. The relationships within the administrative group (49%) (Table 1) and the nursing group (42%) are more dense than between all members of the socialising network, but the socialising ties between the doctors (12%) and between the allied health professionals are less dense (8%). Due to the nature of emergency shift work, breaks need to be staggered so ED doctors may find it more difficult to socialise with each other than would doctors working in other clinical areas.

Individuals with whom to socialise are not very available. Although on average each connected individual is only one to two steps away from each other individual (Degree of separation = 1 to 2, Table 1), they can be up to seven steps away (Maximum degree of separation = 7, Table 1). A very low reciprocity (24%, Table 1) of the socialising ties indicates that there was a low level of agreement between socialising pairs. There was also one isolate in the socialising network (Junior doctor 20).

Some of the staff who appear central in the socialising network are Senior RN 4, and Senior nurses 6, 11 and 15. Nurses from across a range of positions are important in the socialising network. Senior nurse 12 was reported to socialise with 42 other ED staff (in-degree = 42). Many ED staff also reported socialising with Senior nurses 4, 6 and 9, Senior RNs 4, 5 and 7, Junior RNs 1, 2, 3 and 8, and EN 4. Senior RN 4 also reported socialising with 95 other staff members from ED (out-degree = 95).

## Discussion

Despite the ED often being construed as one big team or workforce, communication across the ED can be clearly understood in terms of three professional groups. Interaction in all three networks occurs mainly within professional groups, with Figures 1, 2 &3 showing most ED staff adjacent to others from their own profession. Studies of networks in other hospital settings have shown similar divisions by professional groups[16,26]. Notwithstanding that, there are significant levels of connectedness across all staff in the ED with typically only one to two degrees of separation in all three networks. The problem-solving network had the greatest number of connections amongst staff, followed by the medication-advice seeking network and lastly the socialising network with the fewest connections.

In the problem solving network the ED staff have a high level of reliance on each other for support to resolve problems. Some senior nurses and doctors play a central role in helping most of the other staff in the network. The roles of these key personnel are consistent with findings from a study of communication patterns in a US emergency department where there were clusters of communication around the charge nurse and where senior doctors communicated frequently with each other[27].

The medication advice data illuminate how doctors are very central in giving advice relating to medication decisions and tasks. Doctors' expertise in medication and their role in prescribing likely explains their importance in this network. Consistent with this, the attitudinal data show that ED staff believe that doctors do not often seek advice from nurses about prescribing.

Social tribes, like professional tribes, are split along professional lines. People are closer to and interact more readily with those with whom they identify, trained with, or share practices with, and to whom they are ideologically and attitudinally closer.

Communication processes consume around 80%[5] of the time of health professionals in the emergency department and yet there are few studies of how health professionals communicate or the content of such communication[28]. The importance of such work is evident in the potential implications for the quality of care. Good communication is essential in an ED environment where 12% of errors are attributed to communication problems[29], with failure to seek information for decision making contributing to 28% of errors in an ED, and failure to offer information to support decision making contributing to 20% of errors, and both being prime causes in some cases[30]. Importantly, our results demonstrated how communication was largely divided along professional lines and found most staff agreed that increased communication amongst doctors and nurses would result in fewer medication errors, suggesting that strategies are required to improve communication across professional groups.

This is the first paper to go inside ED networks to provide insights into their characteristics and complexity. Particular strengths of this research are combining network data with attitudinal data and examining three different aspects of communication networks to highlight how they work.

### Limitations

Drawing on data from one ED limits generalisability. Network analysis reveals social and professional structures and characteristics but does not disclose the tenor of the communication or interaction.

### Implications

Knowledge of how professionals relate and the social and professional structures which they form can provide insights of potential benefit to ED staff, their leaders, policymakers and researchers. Training in cross-disciplinary communication and interaction may be beneficial in the ED in order to improve these characteristics.

## Conclusions

EDs are high-load work environments where communication is central to safe and effective care. Our network analyses of communication and socialising patterns revealed a high level of work task support within professional groups as well as strong cross-professional support for solving work-related problems. Medication advice seeking was more centralised within the medical professionals, consistent with their clinical responsibilities. The extent to which socialising occurs between professional groups is low.

## Competing interests

The authors declare that they have no competing interests.

## Authors' contributions

NC, JIW and JB collaborated to conceive the study; NC carried out the study and data analysis and drafted the manuscript. JW participated in the design and coordination of the study and helped to draft the manuscript. JB participated in the design of the study and helped to draft the manuscript. All authors read and approved the final manuscript.

## Pre-publication history

The pre-publication history for this paper can be accessed here:

http://www.biomedcentral.com/1472-6963/9/247/prepub
